# Selective blood–brain barrier penetration and tumor targeting of nitrosylcobalamin in glioblastoma: Pharmacokinetics, tissue distribution, and synergistic activity with trail and temozolomide

**DOI:** 10.18632/oncoscience.654

**Published:** 2026-04-02

**Authors:** Joseph A. Bauer, Annette M. Sysel, Michael J. Dunphy

**Affiliations:** ^1^Nitric Oxide Services, LLC, Akron, OH 44312, USA; ^2^Cleveland Clinic Foundation, Taussig Cancer Center, Translational Hematology and Oncology Research, Cleveland, OH 44195, USA; ^3^Bauer Research Foundation, Inc., Akron, OH 44312, USA; ^4^Division of Mathematics and Sciences, Walsh University, North Canton, OH 44720, USA

**Keywords:** nitrosylcobalamin, glioblastoma, TRAIL, temozolomide, chemotherapy

## Abstract

Background: Glioblastoma multiforme (GBM) remains a lethal brain tumor characterized by poor response to chemotherapy and limited blood–brain barrier (BBB) permeability. Nitrosylcobalamin (NO-Cbl), a nitric oxide (NO)-releasing cobalamin analog, was developed to selectively deliver cytotoxic NO to tumors through the transcobalamin II receptor (CD320).

Methods: NO-Cbl was evaluated across the NCI-60 tumor panel, followed by pharmacokinetic and biodistribution studies in glioblastoma-bearing rats using nitrate and cobalamin quantification in tissues, serum, and cerebrospinal fluid (CSF). Synergistic activity with TRAIL or temozolomide was assessed in human U87 and D54 glioma cells using SRB assays and Chou–Talalay analysis.

Results: NO-Cbl showed broad antitumor activity *in vitro*, with central nervous system tumor cell lines displaying intermediate sensitivity (mean ID50 = 17.6 μM). *In vivo*, NO-Cbl effectively crossed the BBB, with tumor nitrate levels peaking at 20.4 nmol/g at 30 min and remaining elevated at 24 h, confirming tumor-selective accumulation. Serum nitrate exhibited a rapid half-life (4–5 h), while serum and CSF B12 showed slower and variable clearance (21–26 h and 11–12 h, respectively). In glioma cell lines, NO-Cbl synergized with TRAIL and temozolomide (TMZ) (combination index < 1.0), enhancing antiproliferative effects and suggesting potential to overcome resistance mechanisms.

Conclusion: This pilot study demonstrates that NO-Cbl crosses the BBB, accumulates selectively in brain tumor tissue, and synergizes with established and experimental glioblastoma therapies. These findings establish a translational foundation for developing cobalamin-based therapeutics as a novel treatment strategy for glioblastoma.

## INTRODUCTION

Glioblastoma multiforme (GBM) is the most common and aggressive primary brain tumor in adults, characterized by diffuse infiltration, rapid proliferation, and resistance to standard therapies [1]. The median survival for patients with GBM remains less than 15 months despite maximal therapy, which includes surgical resection followed by radiotherapy and concurrent temozolomide chemotherapy [2]. Tumor recurrence is inevitable, and therapeutic resistance is largely attributed to the tumor’s intrinsic heterogeneity, aggressive angiogenesis, and the presence of treatment-resistant glioma stem cells [3, 4].

A major therapeutic obstacle in GBM is the blood–brain barrier (BBB), which impedes the delivery of systemically administered chemotherapeutics [5]. This has prompted the exploration of novel drug-delivery strategies capable of achieving tumor-specific targeting while bypassing the BBB [6, 7].

Cobalamin (vitamin B12) and its analogs have long been explored as selective tumor-targeting agents due to their reliance on the transcobalamin II receptor (CD320) pathway, which is upregulated in proliferating and malignant cells. CD320 is expressed at elevated levels in various malignancies, including GBM, reflecting the high metabolic demand for cobalamin in rapidly dividing tumor cells.

A landmark study by Collins et al. at the Mayo Clinic demonstrated that radiolabeled adenosylcobalamin selectively accumulates in various malignancies, including central nervous system (CNS) tumors, *via* this receptor-mediated mechanism [8]. In a cohort of 30 patients with a range of cancer types, including recurrent glioma and metastatic brain lesions, radiolabeled adenosylcobalamin enabled imaging of tumors, with greater uptake observed in high-grade and metabolically active malignancies. Notably, CNS tumors exhibited delayed but persistent uptake, with optimal visualization at 24 h post-injection, suggesting effective traversal of the BBB and prolonged retention in tumor tissue. The study highlighted the potential of cobalamin analogs as vehicles for diagnostic imaging and therapeutic delivery, reinforcing the translational rationale for using cobalamin-based platforms in the treatment of glioblastoma and other CNS cancers.

Nitrosylcobalamin (NO-Cbl) is a novel nitric oxide (NO)-releasing analog of hydroxocobalamin designed to act as a biologically compatible chemotherapeutic agent [9]. Our earlier investigations established the mechanistic foundation for nitrosylcobalamin (NO-Cbl) as a tumor-selective nitric oxide (NO) donor. In our seminal work [10], NO-Cbl was shown to trigger caspase-8–dependent apoptosis due to release of nitric oxide within tumors. NO-Cbl exhibited broad antiproliferative activity across the NCI-60 human tumor cell line panel, while sparing normal cells. This study provided the first demonstration that a cobalamin-derived compound could serve as a tumor-selective nitric oxide donor capable of exerting cytotoxic effects without systemic toxicity.

Subsequent studies further elucidated the molecular mechanism of action of NO-Cbl via S-nitrosylation of the TRAIL death receptor DR4 [11] in addition to inhibition of NF-κB–mediated survival signaling [12]. Additionally, NO-Cbl demonstrated synergistic antitumor effects with standard of care chemotherapeutic agents [13]. Collectively, these studies confirmed that NO-Cbl delivers bioactive nitric oxide intracellularly, leading to apoptosis and suppression of pro-survival pathways—mechanistic attributes that distinguish it from conventional alkylating or cytotoxic agents.

The therapeutic relevance of cobalamin transport has been further supported by Sysel et al., who demonstrated elevated expression of transcobalamin II (TCII) and its cell-surface receptor CD320 in a broad range of human tumor xenografts [14] and in naturally occurring canine and feline cancers [15]. These findings confirm that the cobalamin uptake pathway is consistently upregulated in proliferating and malignant tissues, reinforcing its potential as both a biomarker of tumor metabolic activity and a vehicle for targeted drug delivery. This body of evidence provided the biological rationale for developing cobalamin analogs such as nitrosylcobalamin (NO-Cbl), designed to exploit this endogenous transport system for selective delivery of nitric oxide–based chemotherapeutics to cancer cells.

Building upon this mechanistic groundwork, the present study was designed to determine whether these antitumor properties could be translated to the central nervous system, where most chemotherapeutic agents fail to achieve therapeutic concentrations due to the blood–brain barrier (BBB). Specifically, we sought to establish proof of concept that NO-Cbl crosses the BBB and selectively accumulates within glioblastoma tissue following systemic administration. To this end, we quantified its pharmacokinetics and tissue distribution in glioblastoma-bearing rats and evaluated its synergistic antiproliferative activity with TRAIL and temozolomide in human glioblastoma cell lines. Together, these studies provide a translational framework for advancing cobalamin-based therapeutics as a novel treatment strategy for glioblastoma.

## RESULTS

### NO-Cbl exhibits broad antitumor activity with enhanced sensitivity in CNS tumors

**Figure 1:**
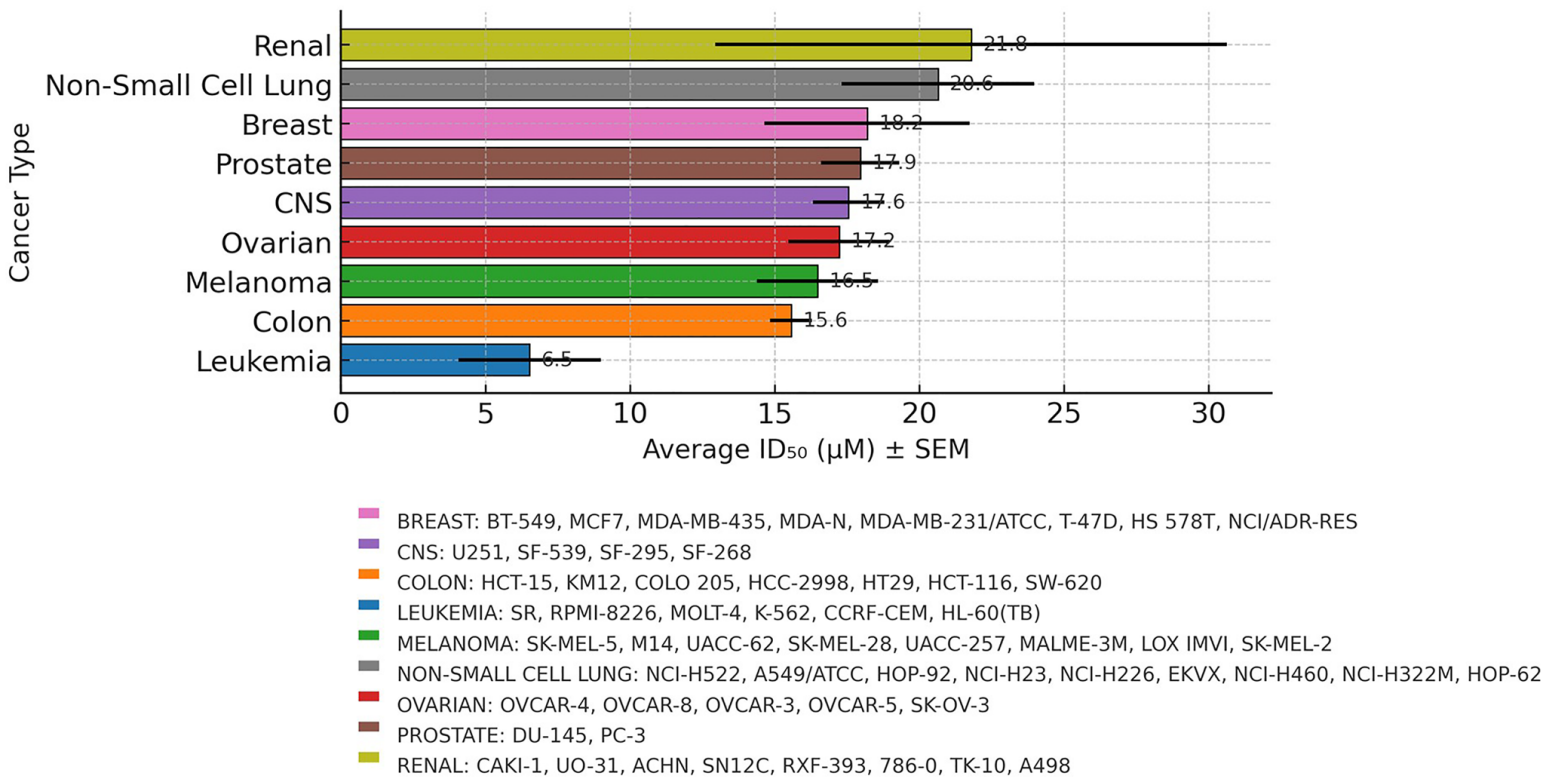
Nitrosylcobalamin (NO-Cbl) inhibits proliferation across NCI60 human tumor cell lines. Mean Inhibitory dose of 50% (ID50) +/− SEM values for NO-Cbl were determined for each major tumor type using the NCI60 cell line panel and sulforhodamine B assays. Renal, non-small cell lung cancer (NSCLC) and breast cancer cell lines were the most resistant, while leukemia cells were the most sensitive. Central nervous system tumor cell lines, highlighted in purple, exhibited intermediate sensitivity.

A comprehensive proliferation screen was performed using the NCI60 human tumor cell line panel, which includes representatives from nine major tumor types. Cell proliferation following NO-Cbl treatment was quantified using the SRB assay, and mean ID_50_ values were calculated for each tumor type (Figure 1).

NO-Cbl demonstrated antiproliferative activity against all tested tumor types, with mean ID_50_ values ranging from 6.5.1 μM to 21.78 μM. Hematological malignancies (leukemia cell lines) were the most sensitive to NO-Cbl, exhibiting the lowest mean ID_50_ value (6.5 μM). CNS tumor cell lines showed intermediate sensitivity (ID_50_ = 17.55 μM) while renal, non-small cell lung cancer, and breast cancer cell lines were relatively more resistant, with mean ID_50_ values above 18 μM.

### Pharmacokinetic assessment of NO-Cbl

#### Tissue nitrate and cobalamin (B12) levels following intraperitoneal administration of NO-Cbl

**Figure 2:**
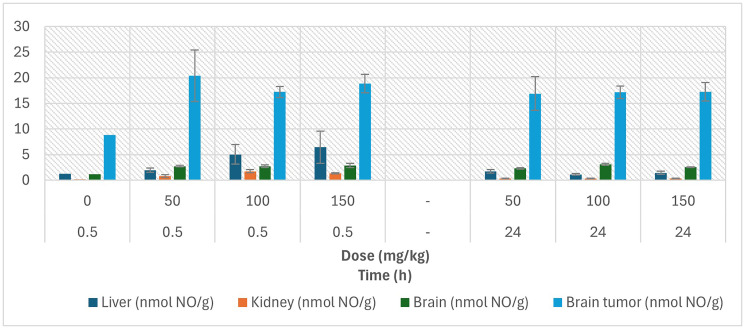
Tissue nitrate and cobalamin levels following intraperitoneal administration of NO-Cbl in rats. Nitrate concentrations (as nmoles NO/g of tissue) measured in liver, kidney, brain, and brain tumor from male rats bearing intracranial glioblastoma xenografts. Animals received a single intraperitoneal bolus of NO-Cbl at 50, 100, or 150 mg/kg. Measurements were taken at baseline (PBS), 30 min, and 24 h post-injection.

To characterize the biodistribution of NO-Cbl and its metabolites, nitrate and cobalamin concentrations were measured in major tissues at 30 minutes and 24 hours following a single intraperitoneal bolus of NO-Cbl (50, 100, or 150 mg/kg) in male rats bearing intracranial glioblastoma xenografts (Figure 2).

At 30 minutes post-injection, nitrate concentrations were elevated in all tissues relative to PBS controls, with the highest levels detected in brain tumor tissue (17–20 nmol NO/g). Liver nitrate increased modestly (1.9–6.5 nmol NO/g across doses), while kidney and brain tissues exhibited smaller elevations (≤2.7 nmol NO/g). By 24 hours, nitrate levels in liver, kidney, and brain declined toward or below baseline, indicating rapid clearance of NO-derived metabolites. In contrast, brain tumor nitrate remained persistently elevated (≈16–17 nmol NO/g) across all doses, demonstrating sustained intratumoral retention and localized NO release within the tumor microenvironment. Partial AUC_0.5–24h_ values, estimated using the trapezoidal rule, ((*C*_0.5_ + *C*_24_)/2) × 23.5 h, confirmed that cumulative nitrate exposure was greatest in tumor tissue, consistent with preferential accumulation of NO species in neoplastic regions.

#### Serum nitrate and cobalamin (serum and CSF) levels following intraperitoneal administration of NO-Cbl

**Figure 3:**
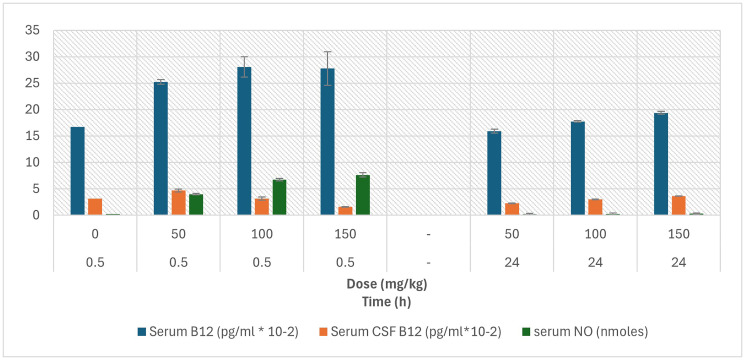
Serum nitrate and cobalamin levels following intraperitoneal administration of NO-Cbl in rats. Serum nitrate, cobalamin, and cerebral spinal fluid (CSF) cobalamin were measured in the same animals and at the same time points and treatment groups as in (Figure 2). Values are expressed as the mean ± standard deviation (*n* = 3 per group).

Systemic pharmacokinetics of NO-Cbl were assessed by measuring serum nitrate and cobalamin concentrations, as well as cerebrospinal fluid (CSF) cobalamin, at 30 minutes and 24 hours following intraperitoneal administration (Figure 3).

At 30 minutes, serum nitrate increased in a dose-dependent manner, rising from 0.23 nmol in PBS controls to 3.97 nmol and 7.63 nmol at 50 and 150 mg/kg, respectively. By 24 hours, nitrate concentrations returned to near baseline (0.21–0.34 nmol). Corresponding partial AUC_0.5–24h_ values ranged from ~49 to 94 nmol·h, consistent with a rapid but transient systemic release of nitric oxide.

Serum cobalamin concentrations increased from 16.7 pg/mL × 10^−^² at baseline to 25–28 pg/mL × 10^−^² at 30 minutes, with sustained but reduced levels (15.9–19.4 pg/mL × 10^−^²) at 24 hours, indicating prolonged circulation of cobalamin species. CSF cobalamin exhibited a modest and variable response: levels peaked at 4.7 pg/mL × 10^−^² at 50 mg/kg and 0.5 h, decreased at higher doses, and returned toward 2–3.6 pg/mL × 10^−^² by 24 hours.

Together, these data indicate that NO-Cbl administration produces a rapid systemic surge in oxidation products of NO with sustained cobalamin exposure, and preferential accumulation of nitrate within tumor tissue, supporting a mechanism of localized NO delivery and retention at the tumor site.

### NO-Cbl potentiates antiproliferative effects of TRAIL and temozolomide in human glioblastoma cell lines

To assess the efficacy of NO-Cbl in combination with established therapeutic agents, U87 and D54 human glioblastoma cells were treated with TRAIL or temozolomide, respectively, with or without NO-Cbl at three increasing concentrations. Cell proliferation was quantified after 72 h using the SRB assay, and results are presented as growth as a percentage of the control.

**Figure 4:**
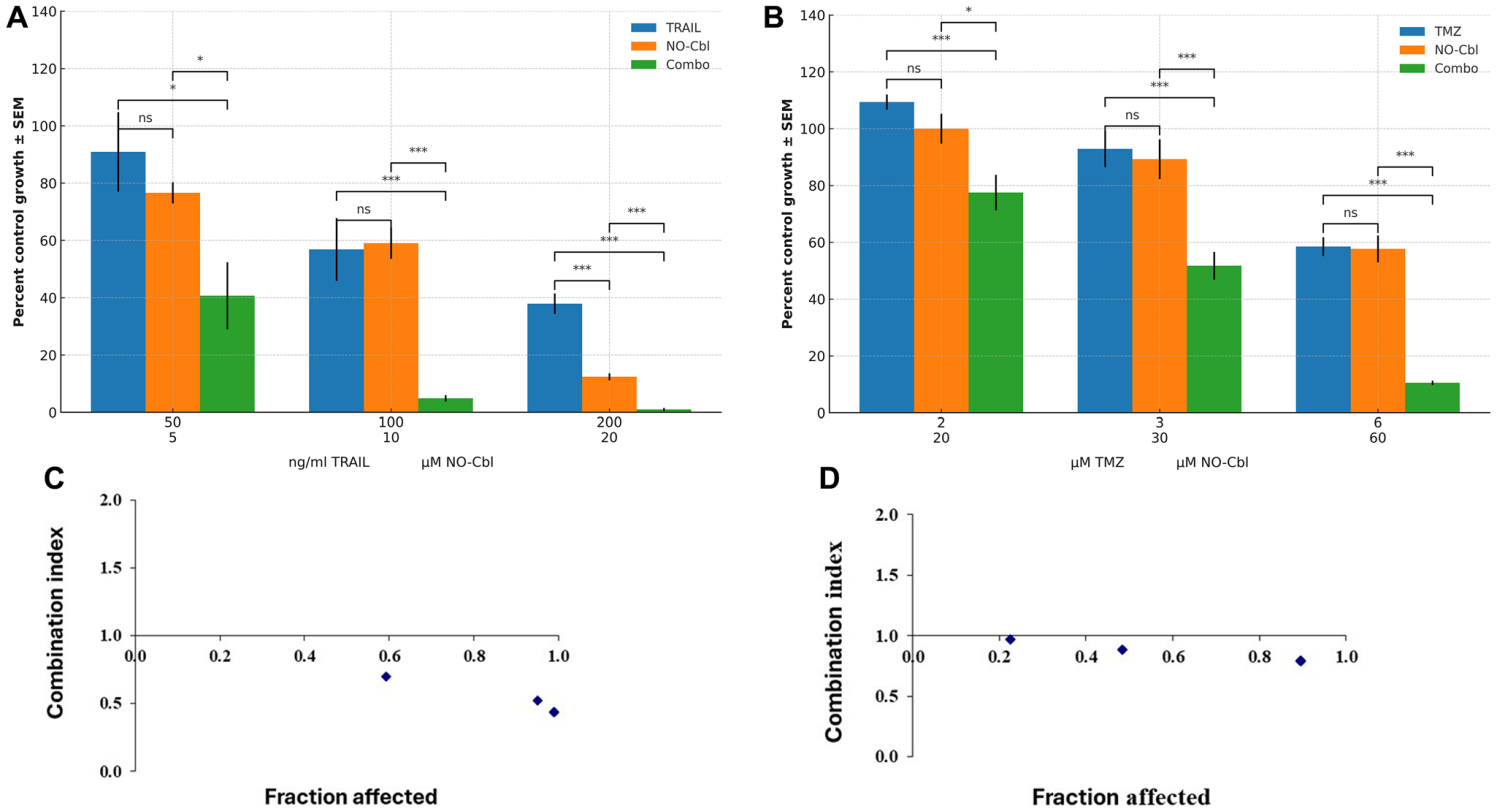
Synergistic inhibition of glioblastoma cell proliferation by NO-Cbl in combination with TRAIL or TMZ. (**A**) Growth of U87 treated with TRAIL, NO-Cbl or their combination and (**B**) D54 cells treated with TMZ, NO-Cbl, or their combination at three dose levels for each treatment. Statistical significance was determined using two-tailed Student’s *t*-tests between treatment groups (TRAIL vs. NO-Cbl, TRAIL vs. Combo, and NO-Cbl vs. Combo for U87; TMZ vs. NO-Cbl, TMZ vs. Combo, and NO-Cbl vs. Combo for D54). Significance levels are denoted as: *p* < 0.05 (^*^), *p* < 0.01 (^**^), *p* < 0.001 (^***^). Combination index plotted against fraction affected for U87 (**C**) and D54 (**D**), treated with TRAIL + NO-Cbl at corresponding combination doses. Combination index values of <1 indicate synergism.

For U87 cells (Figure 4A), TRAIL or NO-Cbl alone produced only modest reductions in cell proliferation at lower doses, with relative growth values remaining above 40% at all but the highest concentrations tested. Notably, the combination of TRAIL and NO-Cbl resulted in a marked, dose-dependent decrease in proliferation, with mean growth reduced to approximately 40%, 5%, and 2% at the low, mid, and high combination doses, respectively. Statistical analysis revealed that the combination treatment significantly inhibited cell growth compared to either agent alone at all doses tested.

Similar results were observed in D54 cells treated with temozolomide and NO-Cbl (Figure 4B). Monotherapy of temozolomide or NO-Cbl produced only partial growth inhibition, whereas combination treatment resulted in substantially lower cell viability, particularly at the mid and high dose levels (mean relative growth reduced to ~50% and ~10%, respectively). All combination treatments showed statistically significant reductions in proliferation relative to single-agent controls.

### Synergy analysis by combination index

The potential for synergy between NO-Cbl and TRAIL or temozolomide was further evaluated using the Chou–Talalay method to determine the CI at each dose (Figure 4C, 4D). For both U87 and D54 cell lines, CI values for all combination doses were less than 1 (range = 0.45–0.97), with the greatest synergy observed at higher fa values. These findings indicate that NO-Cbl acts synergistically with both TRAIL and temozolomide in suppressing glioblastoma cell proliferation.

## DISCUSSION

GBM remains one of the most formidable challenges in neuro-oncology, with standard-of-care treatment offering only modest extensions in survival [16]. The NCI60 screening data demonstrated that NO-Cbl exerts potent antiproliferative activity across multiple tumor types, with CNS tumor cell lines ranking among the most sensitive (average ID_50_ = 17.6 μM). This observation is particularly relevant given the refractory nature of GBM to many conventional agents evaluated through similar screens, including alkylating agents and kinase inhibitors, many of which show diminished activity in CNS models due to poor BBB permeability or non-specific toxicity [17].

Our *in-vivo* pharmacokinetic studies revealed that NO-Cbl crosses the BBB and preferentially accumulates in brain tumor tissue. At 30 min post-injection, levels of nitrate in tumor reached 20 nmol/g, and its concentration remained elevated even at 24 h, while levels in the kidney had rapidly declined. These results contrast sharply with traditional chemotherapeutics such as temozolomide, which display rapid systemic clearance and poor CNS-to-tumor specificity [18]. Furthermore, unlike therapies that require invasive delivery [19, 20] (e.g., convection-enhanced delivery or intrathecal infusion), NO-Cbl achieves effective tumor exposure following simple intraperitoneal administration.

In this study, NO-Cbl enhanced the cytotoxic effects of both TRAIL and temozolomide. Chou–Talalay analysis of effects on U87 and D54 cells confirmed robust synergy (CI <1.0) across multiple effective dose ranges. While temozolomide efficacy is often limited to gliomas with methylated promoter of the gene encoding the DNA-repair enzyme *O*^6^-methylguanine-DNA methyltransferase (*MGMT*), which silences and enhances temozolomide sensitivity [21, 22], NO-Cbl showed activity across both temozolomide-sensitive and -resistant models. Specifically, U87 cells are known to harbor a methylated *MGMT* promoter, rendering them relatively sensitive to temozolomide, whereas D54 cells exhibit an unmethylated *MGMT* promoter, leading to higher MGMT expression and greater temozolomide resistance [23, 24]. Despite this distinction, strong synergy was demonstrated in both cell lines when NO-Cbl was combined with temozolomide, suggesting that NO-Cbl may circumvent MGMT-associated resistance mechanisms, potentially through previously characterized pathways such as caspase-8-mediated apoptosis [10] and inhibition of nuclear factor-kappa B (NF-κB) signaling [12].

The interaction between NO-Cbl and TRAIL signaling offers mechanistic insight into this observed synergy. We demonstrated previously that NO-Cbl induces *S*-nitrosylation of TRAIL receptor, TNF receptor superfamily member 10A (TNFRSF10A, alias TRAILR1), at cysteine residue C336, a post-translational modification that was critical for initiating extrinsic apopotic signaling [11]. Mutation of this residue (C336A) abrogated *S*-nitrosylation and diminished caspase-8 activation, leading to resistance to both NO-Cbl and TRAIL [11]. In our current study, robust synergy was observed between NO-Cbl and TRAIL in both U87 and D54 gliobastoma cells, suggesting that *S*-nitrosylation of TRAILR1 may similarly enhance apoptotic signaling in GBM models. We also demonstrated in earlier work that NO-Cbl did not induce *S*-nitrosylation of TNFRSF10A (alias TRAILR1), indicating receptor-specific modulation of apoptotic sensitivity [11].

Several strategies have been explored to overcome TRAIL resistance in GBM, including the use of histone deacetylase inhibitors, proteasome inhibitors such as bortezomib and microRNA modulation [25]. These agents typically act by downregulating anti-apoptotic proteins, modifying death-inducing signaling complex formation, or altering receptor trafficking. However, they often require high doses or show limited efficacy in glioma stem-like cells, the presumed drivers of recurrence [26]. Notably, TRAIL has been shown to activate compensatory NF-κB signaling in GBM, contributing to therapeutic resistance [25]. In this context, the ability of NO-Cbl to suppress NF-κB activity while concurrently enhancing TRAILR1-mediated apoptosis through *S*-nitrosylation presents a distinct dual mechanism of action. This receptor-specific priming may enable NO-Cbl to convert a non-lethal TRAIL stimulus into a pro-apoptotic response, overcoming a major barrier in the clinical translation of TRAIL-based therapies.

### Strengths and limitations

The major strength of this study lies in its direct demonstration that a cobalamin-based nitric oxide donor can cross the blood–brain barrier and selectively accumulate within intracerebral glioblastoma tissue following a simple intraperitoneal injection. This observation provides experimental validation of the long-hypothesized ability of cobalamin analogs to penetrate the CNS via receptor-mediated transport. The pharmacokinetic and tissue-distribution data generated here provide a framework for optimizing dosing schedules, formulation strategies, and combinatorial regimens involving NO-Cbl.

As a pilot investigation, this work intentionally prioritized distribution and translational feasibility over mechanistic depth. The study used two well-characterized glioblastoma cell lines, U87 (MGMT-methylated, temozolomide-sensitive) and D54 (unmethylated, temozolomide-resistant), to illustrate complementary synergy with TRAIL and temozolomide. While this dual-model approach provided insight into resistance modulation, additional mechanistic exploration using patient-derived xenografts and glioma stem-like populations is warranted. Interspecies differences in cobalamin metabolism and nitric oxide handling also limit direct extrapolation to human pharmacokinetics.

## MATERIALS AND METHODS

### Synthesis of nitrosylcobalamin

Nitrosylcobalamin was synthesized as previously described [9]. Hydroxocobalamin (vitamin B12a) acetate (Hebei Huarong Pharmaceutical Co, Hebei Province, P. R. China) was dissolved in dichloromethane (OmniSolv, EMD Chemicals, Gibbstown, NJ, USA) and exposed to CP grade NO gas (Praxair, Bethlehem, PA, USA) at 150 psi. The reaction proceeded in a closed system within a high-pressure stainless-steel reactor (Parr Instrument Co, Moline, IL, USA). The system was purged daily and evacuated prior to NO exposure. The NO gas was scrubbed prior to entering the system using a stainless-steel cylinder (Midwest Process Controls, Bay Village, OH, USA) containing NaOH pellets. The solid NO-Cbl product was collected following rotary evaporation of the solvent and stored under argon at −80ºC prior to use.

### NCI60 cell line screen and ID50 determination

Nitrosylcobalamin was screened for antiproliferative activity across the NCI60 human tumor cell line panel (Molecular Pharmacology Branch, Frederick National Laboratory for Cancer Research, Frederick, MD, USA) which includes diverse cancer types such as CNS, leukemia, breast, colon, lung, prostate, renal, melanoma, and ovarian [27]. Cells were cultured under standard conditions and seeded in 96-well plates at densities determined by doubling time to reach log-phase growth at the time of drug addition. NO-Cbl was prepared in aqueous buffer and added at five 10-fold serial dilutions (range: 0.01-100 μM). After 48–72 h of continuous exposure, cell viability was assessed using a modified sulforhodamine B (SRB) assay [28]. Absorbance was measured at 515 nm, and growth inhibition (GI) curves were generated to calculate GI50 values (μM) for each cell line and the half-maximal inhibitory dose (ID50).

### Cell culture and drug treatments

Human glioblastoma cell lines U87-MG and D54 were maintained in Dulbecco’s modified Eagle’s medium (Cellgro; Mediatech Herndon, VA USA) supplemented with 10% fetal bovine serum (FBS; Mediatech) and 1% antibiotic-antimycotic (GIBCO BRL, Invitrogen, Carlsbad, CA USA) according to American Type Culture Collection recommendations. Cells were maintained in 5% CO_2_ at 37°C in a humidified incubator. Cells were confirmed as mycoplasma-free using a commercially available kit (MycoAlert; Cambrex Corporation, East Rutherford, NJ, USA). Cells were plated in 96-well plates and treated with NO-Cbl, TRAIL; a generous gift from Avi Ashkenazi (Genentech Inc, South San Francisco, CA, USA) [29], temozolomide (Sigma, St. Louis MO, USA), or their combinations. Treatments were carried out for 192 h, after which cell proliferation was quantified using the SRB assay.

### SRB assay protocol

Cells were harvested using 0.5% trypsin/0.53 mM EDTA, washed, and plated at a density of 2,000 cells per well in 96-well flat-bottom plates in 200 μL of complete medium (RPMI or Dulbecco’s modified Eagle’s medium with 10% FBS and 1% antibiotic-antimycotic). After a 4-h adherence period, drugs were added in serial dilutions. Cells were incubated for 96-144 h depending on their doubling time.

At the endpoint, the medium was removed, and cells were fixed in 10% trichloroacetic acid for 1 h at 4°C. Fixed plates were washed and stained with 0.4% SRB solution for 30 min. Unbound dye was washed away with 1% acetic acid. Protein-bound dye was solubilized using 10 mM Tris base (pH 10.5), and absorbance was measured at 570 nm. Each condition was performed in replicates of eight. Data were normalized to those of untreated controls to calculate the percentage growth inhibition.

### In-vivo pharmacokinetics and tissue distribution

All experimental procedures were approved by the Institutional Animal Care and Use Committee at the Cleveland Clinic Foundation. The animal care facility provided alternating 12-h light and dark cycles with controlled humidity (50–60%) and temperature (21°C to 25°C). Ultraviolet sterilized water and rodent chow were provided throughout the day. Upon arrival, the rats were quarantined and then transferred to an isolated room with a controlled climate.

U87 human glioblastoma cells were inoculated intracerebrally in male athymic nu/nu rats (Rowett Nude; Charles River Laboratories, Wilmington, MA, USA) as previously described [30]. Following tumor cell implantation and confirmation of tumor growth, animals received a single intraperitoneal bolus injection of NO-Cbl (40 mg/mL in THAM pH 7.8) at 50, 100, or 150 mg/kg. Animals were sacrificed at either 30 min or 24 h post-injection (*n* = 3 per group). Blood, CSF, liver, kidney, brain, and brain tumor samples were harvested.

### NO-Cbl quantification

To evaluate NO-Cbl biodistribution tissue and fluid samples were assayed for total nitrate content using a vanadium (III) chloride reduction method coupled with chemiluminescence detection [31, 32]. NO was measured as total nitrate (NO_x_) using the reduction of nitrate and nitrite to nitric oxide gas by 0.4 M vanadium (III) chloride in 1.5 M HCl at 120°C in a custom-built glass impinger (Ace Glass, Vineland, NJ, USA) as previously described [33]. Ten microliters of each sample were injected into the heated VCl_3_ reagent stream, and NO gas released was swept by helium into a Sievers NOA 280i (Sievers Instruments, Boulder, CO, USA) nitrogen oxide analyzer for detection by chemiluminescence. Sample values were calculated against a standard curve generated using known concentrations of potassium nitrate, ranging from 0.1 to 10 μM. All samples were analyzed in quadruplicate. For serum and CSF, nitrate concentrations are reported as nanomoles per milliliter. Tissue values were normalized to wet weight and expressed as nanomoles of NO per gram of tissue. All values are reported as the mean ± standard deviation, with three animals per group per time point.

Serum and CSF cobalamin concentrations were measured using a validated electrochemiluminescence immunoassay, Elecsys System (Roche Diagnostics, Indianapolis, IN, USA) which uses intrinsic factor to bind and determine NO-Cbl concentration.

### Statistical analysis

Data are presented as mean ± standard deviation of triplicate measurements unless otherwise noted. Statistical significance was defined as *p* < 0.05. Combination index (CI) values were calculated using the Chou–Talalay method [34], where CI <1.0 indicates synergism, CI = 1.0 indicates additive effects, and CI >1.0 indicates antagonism. Fraction-affected (fa) values were derived from dose–response curves, and CI *versus* fa plots were used to evaluate combination effects. For pharmacokinetic studies, non-compartmental analysis was performed using time-concentration data collected at 30 min and 24 h following a single intraperitoneal injection of NO-Cbl (50, 100, or 150 mg/kg). The area under the concentration–time curve (AUC_0–24 h_) was calculated using the trapezoidal rule.

## CONCLUSIONS

Future studies will focus on orthotopic validation, longitudinal NO tracking, and dose optimization, as well as integration of mechanistic endpoints such as apoptosis, NF-κB inhibition, and protein nitrosylation within CNS tumor models. Collectively, these findings underscore NO-Cbl’s potential as a clinically translatable agent that combines receptor-targeted BBB penetration with multifaceted anticancer activity.
